# Engineered receptors for human cytomegalovirus that are orthogonal to normal human biology

**DOI:** 10.1371/journal.ppat.1008647

**Published:** 2020-06-19

**Authors:** Jihye Park, Kevin Sean Gill, Ali Asghar Aghajani, Jeremiah Dallas Heredia, Hannah Choi, Adam Oberstein, Erik Procko

**Affiliations:** 1 Department of Biochemistry, University of Illinois, Urbana, Illinois, United States of America; 2 Department of Microbiology and Immunology, University of Illinois College of Medicine, Chicago, Illinois, United States of America; 3 Cancer Center at Illinois (CCIL), University of Illinois, Urbana, Illinois, United States of America; University of Montana, UNITED STATES

## Abstract

A trimeric glycoprotein complex on the surface of human cytomegalovirus (HCMV) binds to platelet-derived growth factor (PDGF) receptor α (PDGFRα) to mediate host cell recognition and fusion of the viral and cellular membranes. Soluble PDGFRα potently neutralizes HCMV in tissue culture, and its potential use as an antiviral therapeutic has the benefit that any escape mutants will likely be attenuated. However, PDGFRα binds multiple PDGF ligands in the human body as part of developmental programs in embryogenesis and continuing through adulthood. Any therapies with soluble receptor therefore come with serious efficacy and safety concerns, especially for the treatment of congenital HCMV. Soluble virus receptors that are orthogonal to human biology might resolve these concerns. This engineering problem is solved by deep mutational scanning on the D2-D3 domains of PDGFRα to identify variants that maintain interactions with the HCMV glycoprotein trimer in the presence of competing PDGF ligands. Competition by PDGFs is conformation-dependent, whereas HCMV trimer binding is independent of proper D2-D3 conformation, and many mutations at the receptor-PDGF interface are suitable for functionally separating trimer from PDGF interactions. Purified soluble PDGFRα carrying a targeted mutation succeeded in displaying wild type affinity for HCMV trimer with a simultaneous loss of PDGF binding, and neutralizes trimer-only and trimer/pentamer-expressing HCMV strains infecting fibroblasts or epithelial cells. Overall, this work makes important progress in the realization of soluble HCMV receptors for clinical application.

## Author summary

Human cytomegalovirus (HCMV) causes severe disease in transplant recipients and immunocompromised patients, and infections in a fetus or neonate are responsible for life-long neurological defects. Cell entry is in part mediated by a trimeric glycoprotein complex on the viral surface, which binds tightly to the host receptor PDGFRα. The soluble extracellular region of PDGFRα can be used as an antiviral agent to potently neutralize the virus *in vitro*. However, PDGFRα ordinarily binds growth factors in the human body to regulate developmental programs, which will limit the *in vivo* efficacy and safety of soluble PDGFRα. Using saturation mutagenesis and selections in human cell culture, mutations in PDGFRα are identified that eliminate off-target growth factor interactions while preserving HCMV binding and neutralization.

## Introduction

Human cytomegalovirus (HCMV; or human herpesvirus 5, HHV-5) is a ubiquitous pathogen that has infected most of the global adult population, and typically causes asymptomatic infections [1,2]. In some cases, individuals will experience mononucleosis-like symptoms, and severe disease can occur in immunocompromised or immunosuppressed adults [3]. HCMV infection may also have an oncomodulatory effect and could be associated with tumor progression, most notably in glioblastoma [4–7], although the mechanistic details remain controversial [8]. However, it is amongst pregnant women and newborns that HCMV has a disproportionate impact on public health [9]. Just over 1 in 200 infants born in the United States are congenitally infected with HCMV, and approximately a fifth of these infants will suffer life-long neurological complications [10,11], including hearing and vision loss, seizures, behavioral disorders, and developmental delays [12–15]. Ideally, infection during pregnancy would be avoided, but this is challenging. The high prevalence of HCMV (especially in families with young children [16,17]), easy transmission through contact with bodily fluids, recurrence of latent endogenous infections [18], reinfection by different exogenous strains [19], and difficulties recognizing asymptomatic infected individuals make controlling virus spread difficult. Furthermore, antivirals have not been approved by the U.S. Food and Drug Administration for routine use in the treatment of congenital HCMV where there are unique concerns regarding toxicity, and drug resistance is widely reported [20]. Faced with this reality, the American College of Obstetricians and Gynecologists does not make any specific recommendations for counseling or treating pregnant women for the prevention of HCMV [21].

It is in this environment that the discovery of an important HCMV receptor, the platelet-derived growth factor receptor α (PDGFRα), has generated excitement towards the development of new therapeutics inhibiting virus-host cell attachment and entry. Two glycoprotein complexes on the HCMV surface drive broad host cell tropism [22]. A pentameric complex of glycoprotein H (gH; UL75), gL (UL115), UL128, UL130 and UL131A mediates entry into epithelial and endothelial cells [23–28], possibly through engagement of neuropilin-2 [29] or the olfactory receptor OR14I1 [30] on the host cell. A trimeric complex of gH, gL and gO (UL74) is sufficient for entry into fibroblasts and is also necessary for entry into pentamer-requiring cell types by promoting membrane fusion [31–33]. Multiple lines of evidence indicate that PDGFRα is the receptor for the trimer. Decreased PDGFRα expression reduces HCMV entry into fibroblasts [34–37]; gO can specifically bind PDGFRα-expressing cells [36]; a quaternary complex of soluble extracellular regions (gH-gL-gO-PDGFRα) can be assembled *in vitro* and the components bind with high nanomolar affinity [29,34]; HCMV strains lacking the trimer fail to enter PDGFRα-dependent cell types to infect cells [36,38]; and the soluble PDGFRα extracellular domain blocks virus entry [29,36,38]. This has led to the soluble domain of PDGFRα or derivative fragments being explored as antivirals [34,36,38], a promising strategy as any mutations in HCMV to escape receptor-based inhibitors would likely decrease binding to the natural receptor and attenuate virulence. Soluble PDGFRα ectodomain effectively blocks virus entry whether applied pre- or post-cell attachment, due to inhibition of both early attachment and fusion steps [38]. Furthermore, soluble PDGFRα inhibits cell entry when only a fraction of glycoprotein trimer is bound and therefore does not require high saturating concentrations; and soluble PDGFRα blocks entry by multiple HCMV strains that express both trimer and pentamer complexes [38].

While appealing, the use of soluble PDGFRα to treat HCMV in humans comes with risk. Indeed, no protein-based viral receptors are currently approved as antiviral drugs by the FDA [39] (although at least one is in a phase II clinical trial for the treatment of COVID-19 by Apeiron Biologics), in part due to safety concerns over interactions with endogenous ligands. By comparison, there are multiple antibody drugs that are specific for viral targets used in the clinic [39]. PDGFRα is an important receptor for platelet-derived growth factors (PDGFs), and regulates cellular proliferation, differentiation and development of multiple tissues during embryogenesis and onwards through adulthood [40]. The PDGF ligands are small polypeptides that covalently associate in disulfide-bonded homo- and heterodimers, which display differing activities towards PDGFRα and its close relative PDGFRβ [41]. Four ligands interact with PDGFRα with high affinity (PDGF-AA, AB, BB, and CC) [42,43] and bind two receptor chains at opposing ends to form a receptor-(ligand)_2_-receptor signaling complex [44,45]. PDGFs compete with the HCMV trimer for PDGFRα binding and block infection [35,36], suggesting that PDGF binding to PDGFRα sterically hinders trimer binding or allosterically modulates the trimer binding interface in a non-productive manner. Treatment with soluble PDGFRα will almost certainly disrupt physiological PDGF-signaling, and the ability of recombinant receptor to bind virions may be diminished by competing endogenous PDGFs. Ideally, mutations in the receptor can be identified that disrupt PDGF interactions while retaining the site or sites engaged by HCMV.

A high resolution crystal structure of PDGF-BB-bound PDGFRβ has illuminated atomic details of ligand-receptor interactions in the family, revealing a predominantly hydrophobic interface at the cleft between the second (D2) and third (D3) extracellular domains of the receptor [44]. However, the binding site for gO is ambiguous. Cryo-electron microscopy suffers from conformational diversity and low resolution [34]; PDGFRα peptide fragment analysis failed to discover any one peptide solely responsible for high affinity binding [38]; and while domain deletions have shown PDGFRα-D3 is important [37], such studies have coarse resolution. The absence of detailed structural information on the trimer-PDGFRα complex means the engineering of HCMV-specific receptors orthogonal to human biology (i.e. receptors that bind virus but do not interact with endogenous human ligands) remains an unsolved challenge.

This challenge is solved through the use of mutagenesis and *in vitro* selection. By tracking the enrichment or depletion of sequence variants in a diverse library using next generation sequencing, the relative phenotypes of thousands of mutations can be simultaneously assessed in a single experiment, referred to as deep mutagenesis [46]. Deep mutational scans have been used to address and engineer specificity in proteins that promiscuously bind multiple ligands [47–49], and methods for deep mutagenesis of membrane proteins expressed in human cells have been optimized [50–52]. By screening through all possible single amino acid substitutions in the D2-D3 domains, we discovered that, unlike PDGF binding, HCMV trimer interactions persist when PDGFRα conformation is disrupted by mutations within the folded core. Particularly relevant to therapeutic design, we also identified PDGFRα surface mutations that retain trimer binding in the presence of high PDGF concentrations. This study brings receptor-based inhibition of HCMV infection closer to realization.

## Results

### A deep mutational scan of PDGFRα D2-D3 domains for trimer binding in the presence of PDGFs

**Fig 1:**
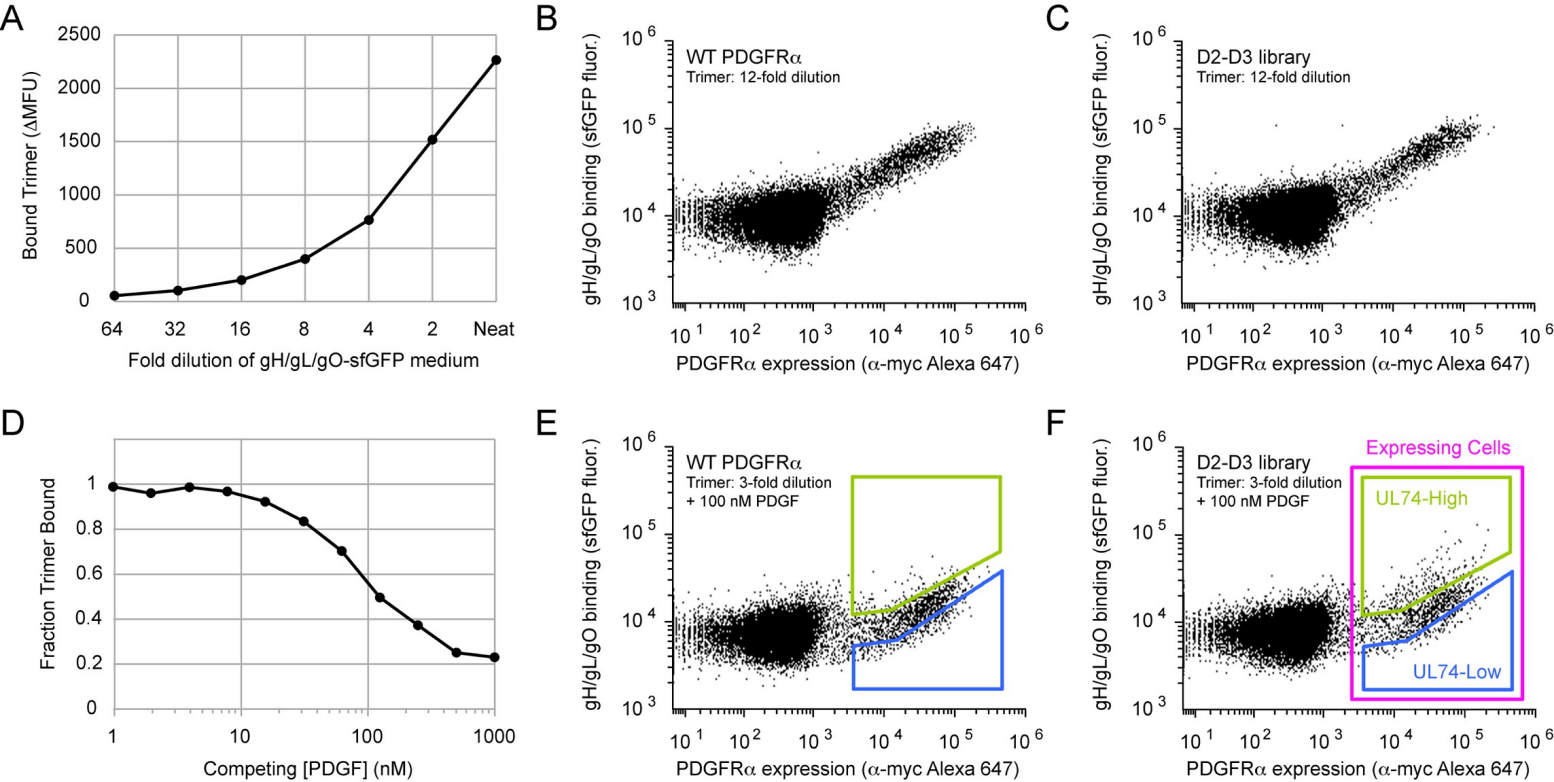
A selection strategy for identifying PDGFRα mutants selective for the HCMV trimer. **(A)** Expi293F cells expressing myc-tagged PDGFRα were incubated with dilutions of gH/gL/gO-sfGFP-containing medium, and binding (shown as ΔMean Fluorescence Units, ΔMFU, in which Mean Fluorescence Units of a negative control without trimer are subtracted as background) was measured by flow cytometry. Neat, no dilution. **(B and C)** Cells were transfected with wild-type (WT) PDGFRα (B) or the D2-D3 SSM library (C) under conditions where typically no more than a single sequence variant is expressed by any given cell. Under these conditions, most cells are negative for PDGFRα expression as determined by anti-myc staining. Cells were incubated with a subsaturating (12-fold dilution) of gH/gL/gO-sfGFP medium and analyzed by flow cytometry. **(D)** Cells were co-incubated with a 2-fold dilution of gH/gL/gO-sfGFP medium and different concentrations of PDGF (an equimolar mixture of PDGF-AA, AB, BB, and CC). The fluorescent signal of gH/gL/gO-sfGFP binding is compared to a sample without competing PDGF. **(E and F)** Cells expressing WT PDGFRα (E) or the D2-D3 SSM library (F) were incubated with a 3-fold dilution of gH/gL/gO-sfGFP medium and 100 nM PDGF (25 nM of each subtype). The choice of a PDGF concentration that does not completely inhibit trimer binding ensured (i) that there remained some positive binding signal for drawing appropriate sort gates, and (ii) that mutations with weak effects will be distinguished from the main population. During sorting of the D2-D3 library, the top 15% of PDGFRα-expressing cells that bind trimer were collected (UL74-High sort, green gate). Simultaneously, the bottom 15% of PDGFRα-expressing cells that bind trimer were also collected (UL74-Low sort, blue gate). For cells expressing WT PDGFRα, the equivalent gates correspond to 4% and 23% of PDGFRα-expressing cells, respectively.

The glycoprotein trimer from HCMV strain TB40/E (BAC4 clone, [53]) was expressed in Expi293F cells, a suspension derivative of human HEK 293. The transmembrane helix of gH was deleted to produce a soluble complex, and the gO subunit, which directly contacts PDGFRα [34,36,54], was fused at its C-terminus to superfolder GFP (sfGFP; [55]) for fluorescence detection. Medium from gH/gL/gO-sfGFP expressing cells was incubated with Expi293F cells transfected with an episomal plasmid encoding PDGFRα. Trimer binding was proportional to PDGFRα surface expression (determined by flow cytometry using antibody staining of an extracellular c-myc epitope tag at the PDGFRα N-terminus; Fig 1A and 1B), and was inhibited by co-incubation with an equimolar mixture of PDGF-AA, AB, BB, and CC (Fig 1D). Medium from cells expressing gO-sfGFP alone, either the wild-type sequence or carrying the mutation C351S (C343S based on numbering of gO from the TB40/E strain) to remove the exposed cysteine that would ordinarily form a disulfide to gL-C144 [56], failed to display high binding signal to PDGFRα positive cells (S1 Fig). The binding signal is therefore dependent on co-expression with gH and gL, suggestive that it is trimer as opposed to monomeric gO that engages the receptor, although this is not explicitly demonstrated.

A single site-saturation mutagenesis (SSM) library of PDGFRα was constructed encompassing all single amino acid mutations in the D2-D3 domains (a.a. D123-E311), and transfected into Expi293F cells under conditions that typically yield no more than one sequence variant per cell, providing a tight link between genotype and phenotype [51]. When incubated with gH/gL/gO-sfGFP medium at subsaturating dilutions, cells expressing the SSM library were surprisingly indistinguishable from cells expressing wild-type PDGFRα (Fig 1B and 1C). Normally, many mutations adversely impact protein activity through destabilization of folded structure or damage to functional sites, and loss-of-function variants tend to dominate naive libraries prior to any selection. That deleterious mutations are not prevalent in the naive SSM library immediately implied that HCMV trimer binding is resistant to most single non-synonymous mutations in the PDGFRα D2-D3 domains.

By comparison, many cells expressing the SSM library displayed higher trimer binding in the presence of PDGFs than cells expressing wild-type PDGFRα (Fig 1E and 1F). There thus appeared to be many PDGFRα mutations that selectively lost PDGF affinity. To identify these mutations, PDGFRα-expressing cells that had elevated HCMV trimer binding in the presence of competing PDGFs were collected by fluorescence-activated cell sorting (FACS). This is referred to as the UL74-High sort (see green gate in Fig 1F). Within the same experiment, cells expressing PDGFRα but displaying low trimer binding were also collected, referred to as the UL74-Low sort (see blue gate in Fig 1F). The UL74 gene encodes gO [31], and the names assigned to the sorted populations correspond to the raw and processed data files deposited in the GEO database [57]. PDGFRα mutants that lose affinity for trimer, or perhaps have enhanced affinity for competing PDGFs, will be preferentially enriched in the UL74-Low sort. PDGFRα mutants that fail to express will be depleted from both sorted populations. Following Illumina sequencing of the naive plasmid library and transcripts from the sorted populations, the enrichment ratios for all 3,780 substitutions in the D2-D3 domains were calculated to define a local mutational landscape (S2A Fig). Data from independent replicates are highly correlated (S2B–S2G Fig), giving confidence in the data’s accuracy.

### PDGF interactions are uniquely sensitive to mutational disruption of folded structure in the D2-D3 domains

**Fig 2:**
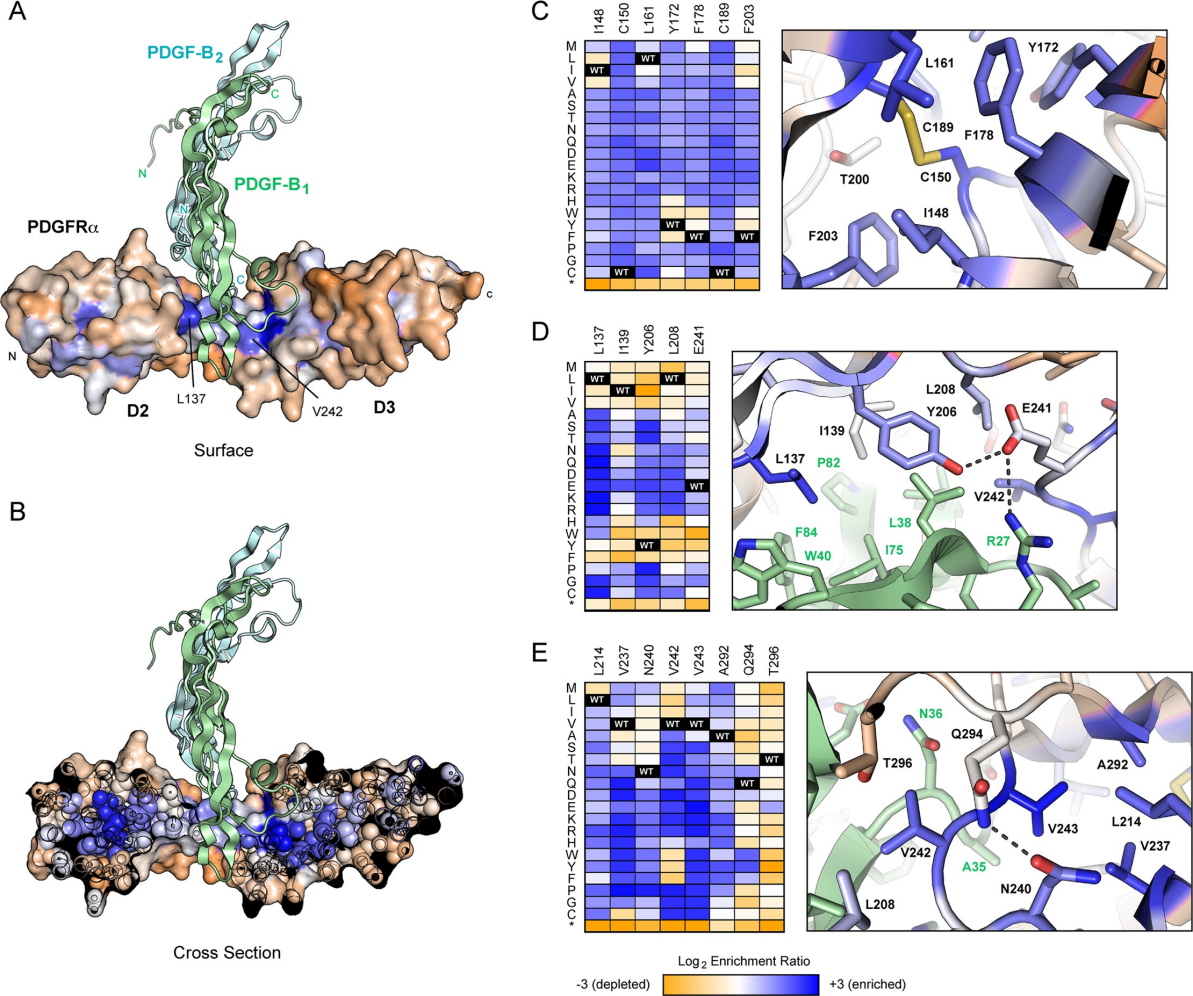
Hot spots in PDGFRα for mutations that increase HCMV trimer binding in the presence of competing PDGFs. **(A)** Model of PDGFRα D2-D3 domains bound to PDGF-BB (protruding subunit B_1_ is pale green, receding subunit B_2_ is pale blue). Conservation scores from the UL74-High sort are mapped to the PDGFRα surface, showing residues where mutations tend to be depleted in orange, and residues where mutations tend to be enriched in dark blue. **(B)** A cross section through PDGFRα highlights that enriched mutations (dark blue) are concentrated in the protein interior/core. **(C-E)** Log_2_ enrichment ratios for individual mutations from the UL74-High sort are plotted from -3 (orange, depleted) to +3 (dark blue, enriched). Amino acid substitutions are indicated on the vertical axis, * is stop codon. The wild-type amino acid is black. Structural views are shown at right, colored as in panel A. Regions shown are the D2 core (C), PDGF-BB interface (D), and PDGF-BB interface and underlying D3 core (E).

Enrichment ratios for nonsynonymous mutations are anticorrelated between the UL74-High and UL74-Low sorts, with few mutations other than premature stop codons being depleted in both selections. Hence substitution mutations in PDGFRα D2-D3 domains suffer little cost to surface localization, even though many mutations will almost certainly destabilize the domain fold. This differs markedly from a prior mutational scan of a multidomain membrane protein expressed in human cells, where nonconservative mutations buried within folded structure prevented protein trafficking to the plasma membrane due to intracellular retention by quality control machinery [50]. When mapping experimental conservation scores to the structure of PDGF-bound PDGFRα (modeled from the crystal structure of PDGF-BB-bound PDGFRβ; PDB ID 3MJG [44]), enriched mutations in the UL74-High sort are found to cluster to buried core positions and the PDGF interface (Fig 2). These include substitutions that create cavities, steric clashes, and/or introduce buried ionizable groups; these mutations are incompatible with biophysical principles governing protein folding and will destabilize folded structure. We hypothesize that PDGFRα mutants with disrupted structure have therefore preferentially lost affinity to PDGFs, thereby reducing competition and enhancing binding of the HCMV trimer. The epitope bound by the HCMV trimer is ambiguous from the data (S3 Fig), but must be at least partially independent of proper D2-D3 conformation, although partial structure may remain. This is consistent with either key contacts to the HCMV trimer residing on linear PDGFRα segments and loops, similar to how many antibodies recognize conformation-independent epitopes, or with favorable interactions to the HCMV trimer being distributed over a broad surface (possibly even beyond the D2-D3 domains) such that localized disruptions are tolerated. Libraries encompassing double or higher order PDGFRα mutations may further resolve details of the binding mechanism. While this illuminates aspects of PDGFRα/trimer recognition, using misfolded, destabilized PDGFRα variants to selectively neutralize HCMV is fraught with risks, including toxicity from non-specific interactions and protein aggregation due to the exposure of hydrophobic residues.

### Surface mutations at the PDGF binding site favor PDGFRα specificity towards the HCMV trimer

The PDGFRα mutational landscape for high trimer binding in the presence of competing PDGF reveals key surface residues as hot spots for enriched mutations, especially PDGFRα residues L137, L208 and V242 that contact the protruding PDGF subunit, and Y206 which packs between the protruding and receding PDGF subunits (Fig 2D and 2E). Mutations to these residues will disrupt the PDGFRα-PDGF interface, especially by the addition of polar substitutions that invert the chemical properties of the highly hydrophobic binding site, while HCMV trimer interactions persist. Overall, enrichment of mutations for increased HCMV trimer binding in the presence of competing PDGFs is highly correlated to the mutated residue’s connectivity within the D2-D3 core or across the PDGFRα-PDGF interface (S4 Fig), emphasizing the selective importance of PDGFRα conformation and surface contacts in the D2-D3 cleft for high affinity PDGF interactions.

**Fig 3:**
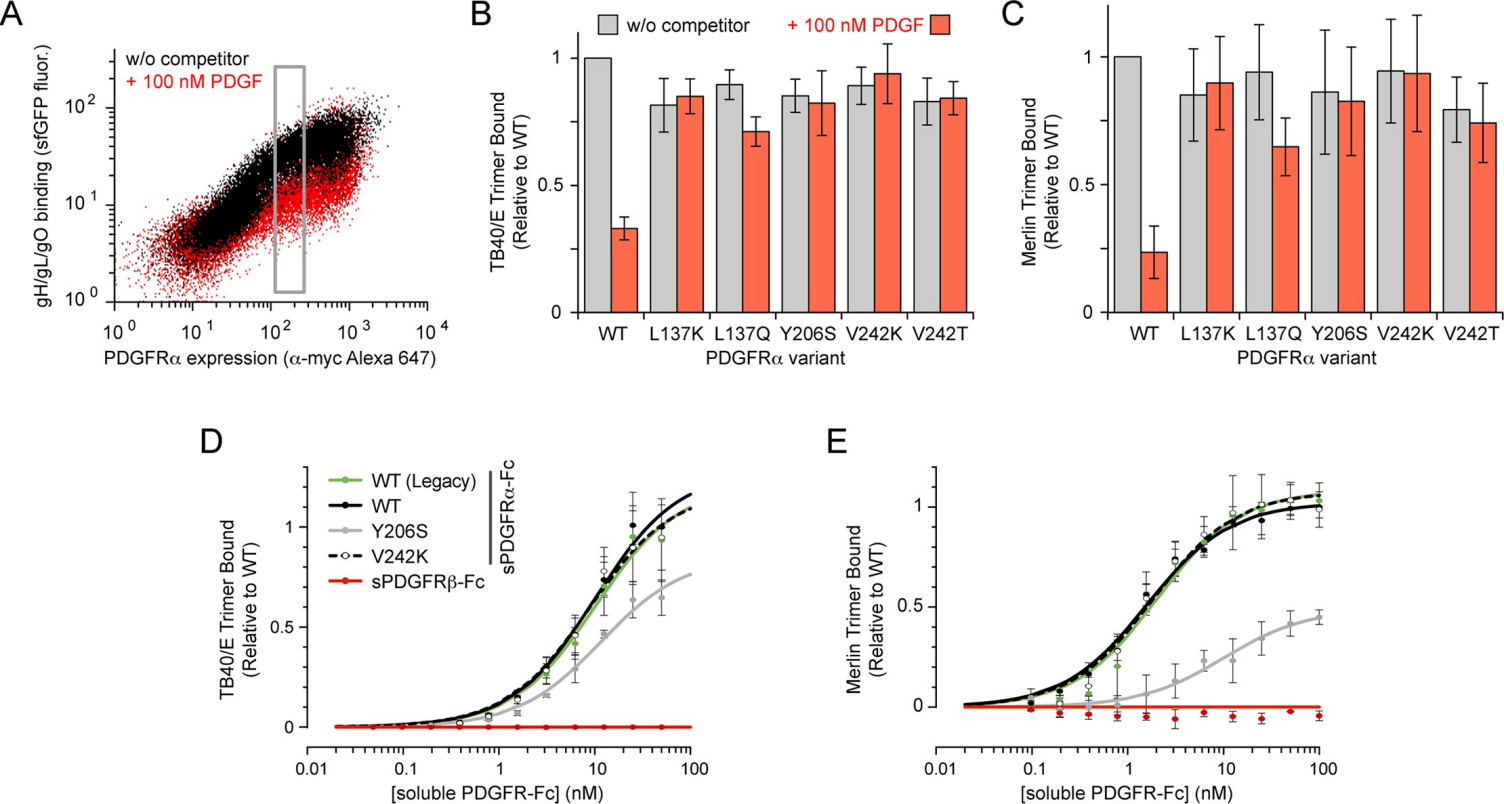
Validation of targeted mutants in PDGFRα that specifically block PDGF competition. **(A)** Expi293F cells expressing wild-type PDGFRα were co-incubated without (black) or with (red) competing PDGFs, and binding of soluble TB40/E trimer (1/3 dilution of medium containing gH/gL/gO-sfGFP) was assessed by flow cytometry. Cells were gated (grey box) to compare binding (measured as ΔMFU) at equivalent receptor expression levels. **(B)** Binding of soluble TB40/E trimer to PDGFRα variants. Cells were analyzed as outlined in panel A. Data are mean ± SD, n = 3. **(C)** Cells expressing PDGFRα variants were incubated with Merlin gH/gL/gO-sfGFP containing medium (1/3 dilution) in the absence (grey) or presence of PDGFs (red). Cells were analyzed as outlined in panel A. Data are mean ± SD, n = 4. **(D)** Expi293F cells were transfected with gL, gO, and full-length gH from strain TB40/E. Binding of sPDGFR-Fc to HCMV trimer anchored at the cell surface was measured by flow cytometry. Data are mean ± SD, n = 2 (sPDGFRβ-Fc), 3 (WT-Legacy and Y206S), 4 (V242K), or 5 (WT). **(E**) Binding of sPDGFR-Fc to cells expressing membrane-anchored trimer from HCMV strain Merlin. Data are mean ± SD, n = 2 (sPDGFRβ-Fc), or 3 (WT-Legacy, WT, Y206S, and V242K).

Five mutations on the PDGFRα surface (L137K, L137Q and Y206S in D2, and V242K and V242T in D3, chosen based on their high enrichment in the UL74-High sort and covering multiple surface positions) were validated as having desirable binding properties by targeted mutagenesis. As predicted from the deep mutational scan, these PDGFRα variants maintain near wild-type levels of binding to soluble HCMV trimer from the TB40/E strain, yet have diminished sensitivity to the addition of competing PDGFs (Fig 3A and 3B), demonstrating successful engineering of specificity towards the viral target. Since the mechanism of trimer binding to receptor has not been resolved at atomic or even residue-level resolution, it remains uncertain whether the viral genome could mutate to distinguish wild-type from engineered PDGFRα. This concern is partially alleviated by the observation that the engineered PDGFRα mutants maintain high binding to soluble HCMV trimer from the Merlin clinical strain [58] (Fig 3C), which has relatively high natural sequence variation compared to TB40/E (79% identity between glycoproteins O). Variation is particularly high in the N-terminus of glycoprotein O where important interactions to PDGFRα reside [54].

Fusion of soluble proteins to immunoglobulin Fc confers multiple desirable properties for a therapeutic [59]. Multimerized Fc chains impose avidity for enhanced apparent affinity, and Fc moieties are engaged by multiple receptors to enhance serum half-life or evoke effector functions, including complement activation and antibody-dependent cell-mediated cytotoxicity [59]. Soluble PDGFRα fused to the Fc region of human IgG1 was shown to bind HCMV trimer when first identified as its receptor [34], and the neutralization properties of Fc-fused soluble PDGFRα (sPDGFRα-Fc) have been explored since in greater detail [38]. These prior studies used commercially supplied sPDGFRα-Fc featuring a random linker of mixed amino acids that connects to the upper hinge of IgG1, upstream of Cys-220 that would ordinarily form a disulfide to the antibody light chain (S5A Fig). The nature of this Fc fusion may cause manufacturing liabilities if Cys-220 is exposed and free, and we refer to this construct as “legacy” sPDGFRα-Fc. We designed an alternative sPDGFRα-Fc construct that includes the PDGFRα signal peptide (a.a. 1–23), ectodomain (a.a. 24–524), a GGGS linker, and finally human IgG1 Fc beginning at Asp-221 (S5A Fig). Both sPDGFRα-Fc constructs bind membrane-localized HCMV trimer with equal affinity (Fig 3D and 3E), and subsequent studies focused exclusively on our redesigned fusion protein.

Soluble PDGFRα-Fc was expressed in Expi293F culture, and was isolated by affinity capture and size exclusion chromatography (SEC) to very high purity (S5B and S5C Fig). The purified protein is monodisperse by SEC, and the final yield is high (> 25 mg / L of transfected culture without any optimization). Soluble PDGFRα-Fc V242K was stressed for 7 days at 40°C, pH 8.5, to promote asparagine deamidation and protein denaturation [60], yet remained mostly stable based on the SEC profile (S6 Fig). A harsher stress for 14 days at 40°C, pH 5.5, to promote asparagine isomerization and protein denaturation [60] caused sPDGFRα-Fc V242K to form soluble aggregates (S6 Fig). We conclude that the protein has moderate stability and is suitable for further manufacturing development.

Two PDGFRα mutations, Y206S and V242K, were explored as Fc-fused soluble orthogonal receptors. Binding of the soluble receptors was measured towards HCMV trimer expressed at the cell surface, with gL and gO subunits tagged at their C-termini with short peptide epitopes, and gH expressed as full-length protein with its native transmembrane domain. The Y206S mutant had reduced binding to HCMV trimer compared to wild-type receptor, but sPDGFRα-Fc V242K bound HCMV trimer from Merlin and TB40/E strains with equal low nanomolar affinity (Fig 3D and 3E). Aspects of these experiments were replicated with independent protein preparations (S7A Fig). Furthermore, fusions of wild-type and V242K sPDGFRα with the Fc region of IgG3 also bound similarly to trimer from the TB40/E and Merlin strains (S7B and S7C Fig). Compared to IgG1, IgG3 has lower affinity for the neonatal Fc receptor (FcRn), which is associated with reduced serum half-life and placental transfer [61,62]. We speculate that these features may be advantageous in the treatment of pregnant women during acute HCMV infection to achieve a lower dose in the developing fetus, thereby further addressing safety concerns.

**Fig 4:**
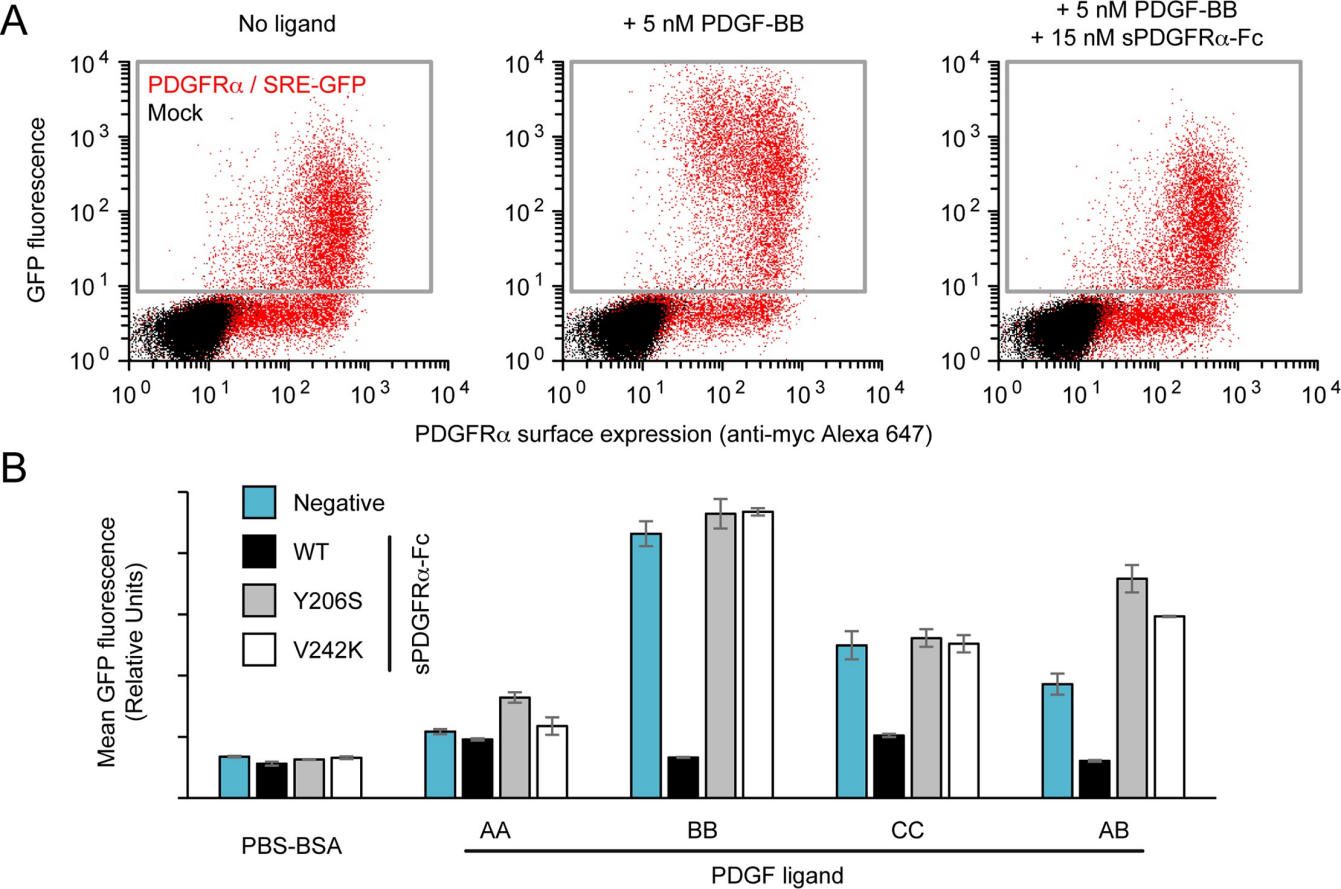
Soluble PDGFRα variants that no longer inhibit PDGF signaling. (A) Flow cytometry analysis of mock-transfected Expi293F cells (black), or cells transfected with a plasmid driving myc-tagged PDGFRα expression and carrying a SRE-regulated GFP reporter (red). Signaling activity was quantified by measuring mean GFP fluorescence (vertical axis) of responsive cells in the grey gate. Background PDGFRα signaling is at left, PDGF-BB stimulated activity is center, while sPDGFRα-Fc is shown to inhibit PDGF-BB stimulation at right. (B) Stimulation of transfected Expi293F cells with 5 nM PDGF ligands. PDGF ligands were pre-incubated with 0.2% BSA (negative, blue) or with a 3-fold molar excess of wild-type sPDGFRα-Fc (black), sPDGFRα-Fc Y206S (grey), or sPDGFRα-Fc V242K (white). Data are mean ± SD, n = 3 independent replicates.

To further validate that the engineered receptors have lost growth factor interactions, we assessed their ability to inhibit PDGF signaling. Expi293F cells were transfected with a reporter plasmid encoding wild-type PDGFRα and a destabilized fluorescent protein (EGFP-PEST) [63] under the control of a serum response element (SRE) [64]. Treatment with PDGFs upregulates EGFP-PEST expression, which is inhibited by wild-type sPDGFRα-Fc acting as a decoy receptor (Fig 4). As intended, both sPDGFRα-Fc Y206S and V242K failed to inhibit PDGF signaling (Fig 4B). However, we also noticed that the engineered receptors, in particular sPDGFRα-Fc Y206S, unexpectedly enhanced the response to PDGF-A ligands. We suspect this is due to a generic ‘carrier protein’ effect (PDGF ligands and their receptors are highly hydrophobic [44] and are generally formulated with a carrier such as serum albumin), though we are unable to conclude this with certainty, and non-specific gamma globulin has no such effect (S8 Fig). This observation may require future investigation.

### Orthogonal PDGFRα-based receptors targeting HCMV trimer potently neutralize virus

The two representative orthogonal receptors, PDGFRα Y206S and V242K, were evaluated for their efficacy to neutralize virus infection. HCMV trimer-only lab strain AD169 [25,65] and trimer/pentamer-expressing clinical strain TB40/E [53] were grown on PDGFRα-positive MRC-5 fibroblasts and PDGFRα-negative ARPE-19 epithelial cells, respectively. Three cell lines were infected by both HCMV strains: MRC-5, ARPE-19, and ARPE-19RA which are stably transfected with PDGFRα to confer susceptibility to AD169 [37].

**Fig 5:**
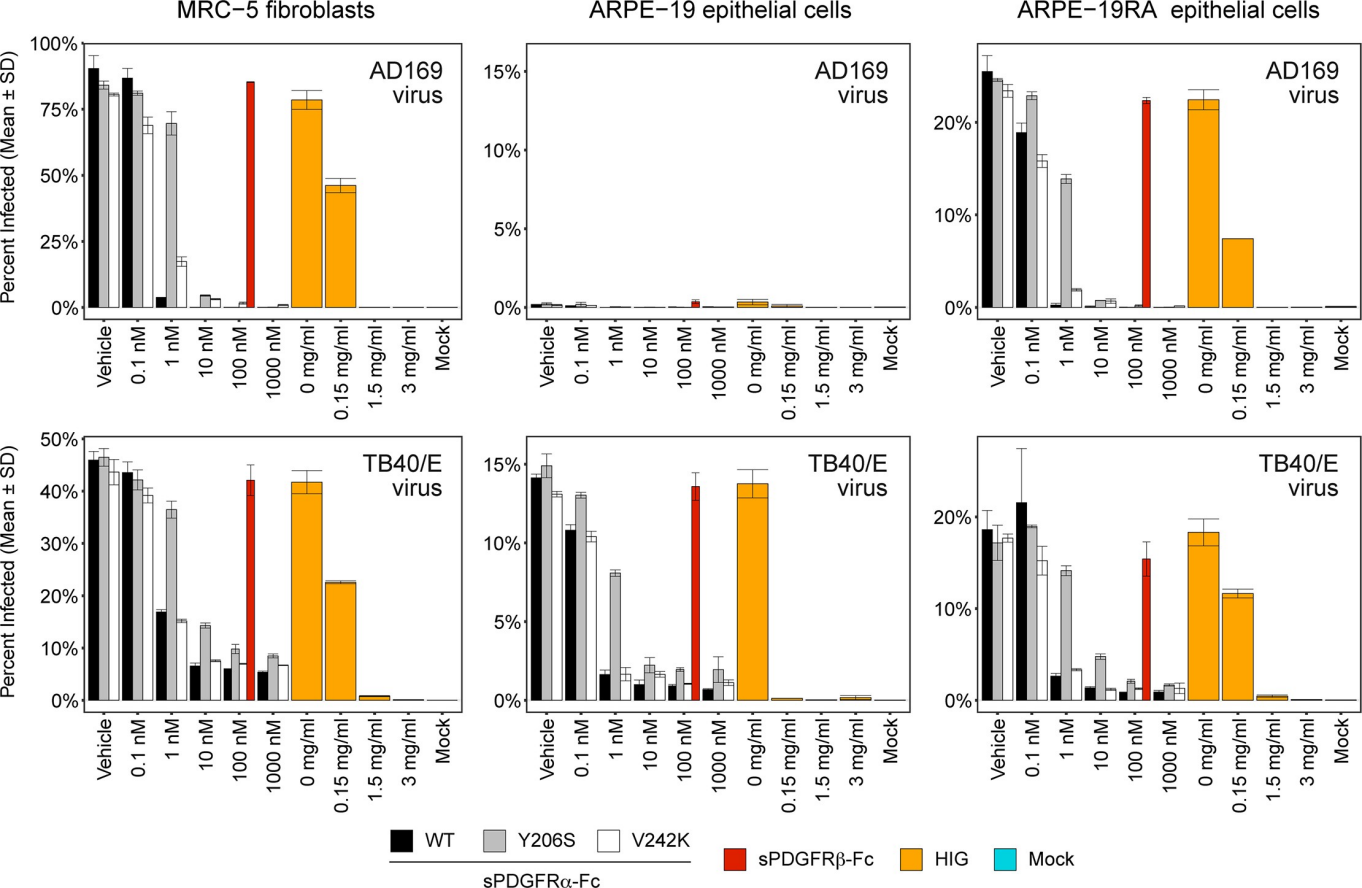
Soluble orthogonal receptors for HCMV trimer neutralize virus. MRC-5 (left), ARPE-19 (middle) and ARPE-19RA (right) cells were infected with HCMV strains AD169 (grown in MRC-5; top) and TB40/E (grown in ARPE-19; bottom). Infection by the trimer-only AD169 strain is restricted to PDGFRα-positive MRC-5 and ARPE-19RA lines. Viral inoculum was pre-incubated with sPDGFRα-Fc WT (black), sPDGFRα-Fc Y206S (grey), sPDGFRα-Fc V242K (white), sPDGFRβ-Fc (at a single concentration of 100 nM; red), or hyperimmune globulin (HIG; orange) prior to adding to cells. Data represent mean ± SD, n = 2 independent titrations.

Soluble PDGFRα-Fc potently neutralized both HCMV strains at nanomolar concentrations (Fig 5). Neutralization by orthogonal sPDGFRα-Fc V242K was mostly indistinguishable from wild-type receptor and nearly complete at 1 to 10 nM (Fig 5), demonstrating that virus binding and neutralization can indeed be successfully separated from growth factor interactions by just a single amino acid substitution. Soluble PDGFRα-Fc Y206S also neutralized but with reduced potency, consistent with the biochemical binding studies. However, neutralization of trimer/pentamer-expressing TB40/E remained incomplete even at high sPDGFRα-Fc concentrations. Hyperimmune globulin (sold under the trade name Cytogam by CSL Behring for prophylactic treatment of transplant recipients) completely neutralizes HCMV, albeit at high concentrations. It is unclear if residual pentamer-mediated infection in the presence of high sPDGFRα-Fc would in any way limit therapeutic or prophylactic efficacy in an *in vivo* setting. Depletion of HCMV-positive patient sera with either trimer or pentamer severely diminishes neutralization of epithelial cell infection [66], suggesting that targeting both glycoprotein complexes can give synergistic effects and is worth further exploration.

## Discussion

Ideas surrounding the use of soluble virus receptors as decoys to treat or prevent infection have a long history [67–70]. Soluble PDGFRα as a prophylactic or treatment for HCMV is particularly promising due to exceptionally tight affinity for glycoprotein trimer and its ability to neutralize both trimer- and pentamer-mediated host cell entry. Here, we have shown how deep mutagenesis can inform the engineering of PDGFRα variants that maintain tight virus binding but have lost growth factor interactions, and are thereby orthogonal to normal human signaling pathways. This is anticipated to improve both efficacy and safety *in vivo*.

There are no simple, tractable animal models for investigating HCMV infection. Two highly relevant models are the study of rhCMV in rhesus macaques [71] or the engraftment of human tissues into immune-deficient mice [72–74], but these are non-trivial and require specialized expertise. However, it might be possible to test biocompatibility and pharmacokinetics of the orthogonal receptors independent of infection in small animal models. For instance, murine and human PDGF ligands and receptors share high identity (> 85%, with all key binding site residues of PDGFRα conserved), and one standard assessment of purified human PDGF ligands is to measure mitogenic activity towards mouse fibroblast lines [75,76]. This suggests the human and mouse PDGF ligands and receptors will cross-react, though this has not been quantitatively characterized. Mice, especially strains expressing human FcRn for human-like serum stability of Fc fused protein [77,78], may therefore be a suitable model for assessing safety during future development.

The binding site for HCMV gO on PDGFRα is uncertain from the deep mutational scan, however the data unambiguously shows that binding is independent of properly folded structure in the PDGFRα D2-D3 domains. Deletion studies have show that the D3 domain is essential for trimer-mediated HCMV entry [37], although no specific hot spot residues for mediating physical binding have been defined. It is possible that gO makes critical contacts to domains D4 or D5 that are outside the mutagenized region in the PDGFRα D2-D3 library, or simply that negative effects of single amino acid substitutions in the D2-D3 domains are too small to cause an observable decrease in binding to HCMV trimer by flow cytometry. Determining how gO binds PDGFRα at atomic resolution will be important for speculating on the likelihood of HCMV acquiring mutations to distinguish and escape from orthogonal receptors.

In our own prior studies, deep mutagenesis of human membrane proteins was used to understand how sequence relates to folding based on surface localization, as misfolded sequences were retained intracellularly due to quality control machinery [50,51,79]. However, PDGFRα is an exception, and nearly every missense mutation still reaches the plasma membrane, including those that are incompatible with the native folded structure (for example, mutations that disrupt disulfides, create cavities, impose steric clashes, or introduce buried charges). The structural organization of PDGFRα as a series of loosely connected β-sandwich domains may facilitate surface trafficking of folding mutants, whereas previous studies have focused on membrane proteins with complex tertiary and quaternary structures.

Finally, deep mutagenesis has been extensively applied to viral surface proteins based on virus replication and propagation in tissue culture as the selection, often in the presence of neutralizing antibodies to discover principles of immune recognition and escape [80,81]. However, it is by decoupling the sequence library from a virus genome [52] that we have been able to determine a mutational landscape of a virus receptor. The principles and approach outlined here can be easily adapted for the engineering of other viral receptors for improved efficacy and safety to treat or prevent infections. Indeed, the same methods we optimized for the development of a HCMV antiviral were rapidly repurposed for engineering soluble decoy receptors that bind with high affinity to the spike of SARS coronavirus 2, a zoonotic pathogen that recently spilled over to the human population and has profoundly impacted world events [82]. Receptor-based antiviral drugs may be especially desirable for emerging zoonotic viruses or viruses with high sequence diversity, as they potentially have broader specificity than monoclonal antibodies to target all virus strains that utilize a common receptor.

## Materials and methods

### Plasmids

Human PDGFRα isoform 1 (GenBank NM_006206.4) was cloned in to the NheI-XhoI sites of pCEP4 (Invitrogen) with a N-terminal HA leader (MKTIIALSYIFCLVFA), myc-tag, linker (GSPGGASG), and followed by the mature polypeptide (a.a. 24–1,089). For measuring signaling activity, a minimal promoter under the control of tandem serum response elements was subcloned from SRE reporter vector_559 (Addgene # 82686) [64], a GFP-PEST fluorescent reporter [63] was inserted at the AscI site, and the entire reporter cassette was inserted at the NruI site of pCEP4-myc-PDGFRα by Gibson assembly. As a negative control, human PDGFRβ isoform 1 (GenBank NM_002609.3) was similarly cloned between the NheI-XhoI sites of pCEP4 with a N-terminal HA leader, myc-tag, and linker that connects to the mature protein (a.a. 33–1,106). No interactions between PDGFRβ and HCMV trimer were observed. Soluble PDGFRα-Fc was cloned in to the NheI-XhoI sites of pcDNA3.1(+) (Invitrogen), and encompassed PDGFRα a.a. 1–524, a short connecting linker as outlined in S5A Fig, and the C-terminus of human IgG1 (GenBank KY432415.1) beginning at either C220 or D221 as described in Results. Alternatively, PDGFRα a.a. 1–524 were fused via linker GGGS to D221-K447 of human IgG3 (GenBank P01860.2; this is an R435 allele with reduced binding to FcRn). Synthetic human codon-optimized gene fragments (Integrated DNA Technologies) for HCMV gO (GenBank ABV71596.1 for TB40/E strain, AJY56739.1 for Merlin strain) were genetically fused at the C-terminus to superfolder GFP [55] (for detection of soluble HCMV trimer binding to PDGFRα-positive cells) or to an 8-histidine tag (for expression of membrane-tethered HCMV trimer), and were ligated in to the NheI-XhoI sites of pcDNA3.1(+). Codon-optimized gene fragments for full-length HCMV gH (GenBank ABV71597.1 for TB40/E strain, YP_081523.1 for Merlin Strain) were cloned in to the NheI-XhoI sites of pcDNA3.1(+) for expression of membrane-tethered HCMV trimer. For production of soluble trimer, the extracellular region of gH with the leader peptide (a.a. 1–717 for TB40/E, a.a. 1–716 for Merlin) was subcloned with a 8-histidine tag. Codon-optimized synthetic genes for gL (GenBank ABV71629.1 for TB40/E strain, YP_081555.1 for Merlin) were cloned with C-terminal FLAG tags in to the NheI-XhoI sites of pcDNA3.1(+). Targeted mutations were made by overlap extension PCR. All plasmids were sequence verified (ACGT, Inc) and are deposited with Addgene.

### Cells and viruses

Expi293F cells (ThermoFisher) are a suspension culture derivative of HEK293, and were cultured in Expi293 Expression Medium (ThermoFisher) at 125 rpm, 8% CO2, 37°C. MRC-5 embryonic lung fibroblasts and ARPE-19 adult retinal pigment epithelial cells were from the American Type Culture Collection and were grown at 37°C, 5% CO2, in DMEM supplemented with 10% fetal bovine serum, 1 mM sodium pyruvate, 2 mM glutamax (Gibco), 10 mM Hepes pH 7.4, 0.1 mM MEM Non-Essential Amino Acids (Gibco), 100 units/ml penicillin G, and 100 μg/ml streptomycin sulfate. ARPE-19 ectopically expressing PDGFRα (“ARPE19-RA” cells) were generated by lentiviral transduction with pLV-EF1a-PDGFRα-IRES-PURO, generously provided by Kai Wu (Princeton University). HCMV virus stocks were prepared by electroporating purified bacmid DNA into either MRC-5 fibroblasts (AD169; [65]) or ARPE-19 retinal pigment epithelial cells (TB40/E, clone BAC4; [53]). Stocks were amplified twice by infecting cell monolayers in 15 cm tissue culture plates followed by 850 cm^2^ roller bottles. Virions were concentrated 100× by centrifugation through a 20% sorbitol cushion, resuspended in phosphate-buffered saline (PBS) containing 1% BSA and 7% sucrose, and frozen at -80°C. Virus stocks were titered on MRC-5 and ARPE-19 cells by Immediate-Early Protein 1 (IE1) fluorescent focus assay [83].

### Flow cytometry analysis of soluble HCMV trimer binding to receptor

Soluble HCMV trimer was prepared by transfecting Expi293F cells at a density of 2 × 10^6^ / ml with 400 ng pcDNA3-gO-sfGFP, 400 ng pcDNA3-gH-8his and 400 ng pcDNA3-gL-FLAG per ml culture using Expifectamine (ThermoFisher). Transfection Enhancers (ThermoFisher) were added 18 h later, and 4 days post-transfection the culture was centrifuged (1,000 × g, 10 minutes, followed by a second spin of the supernatant at 21,000 × g, 5 minutes). The medium supernatant was stored at -20°C and used directly in binding experiments. Expi293F cells at 2 × 10^6^ / ml were transfected with plasmids encoding receptors (500 ng DNA per ml of culture) using Expifectamine. 24 h post-transfection the cells were washed with PBS supplemented with 0.2% bovine serum albumin (PBS-BSA), incubated for 30 minutes on ice with anti-myc Alexa 647 (clone 9B11, 1/250 dilution; Cell Signaling Technology) and the indicated dilutions of gH/gL/gO-sfGFP-containing medium, washed twice with PBS-BSA, then analyzed on a BD LSR II flow cytometer. For competition assays, washed cells were pre-incubated with PDGF ligands (R&D Systems) for 2 minutes on ice prior to addition of gH/gL/gO-sfGFP-containing medium.

### Deep mutagenesis

The D2-D3 domains (a.a. D123-E311) within plasmid pCEP4-myc-PDGFRα were mutagenized by overlap extension PCR [84] using primers with degenerate NNK codons. The plasmid library was transfected in to Expi293F cells using Expifectamine under conditions previously shown to typically give no more than a single coding variant per cell; 1 ng coding plasmid was diluted with 1,500 ng pCEP4-ΔCMV carrier plasmid per ml of cell culture at 2 × 10^6^ / ml, and the medium was replaced 2 h post-transfection. The cells were collected after 24 h, washed with ice-cold PBS-BSA, and incubated for 2 minutes on ice with PDGF-AA, -AB, -BB, and–CC (25 nM of each after addition of soluble trimer-containing medium) and anti-myc Alexa 647 (clone 9B11, 1/250 dilution; Cell Signaling Technology). Medium from cells expressing gH/gL/gO-sfGFP was then added to a final dilution of 1/3, and the cells were incubated on ice for 20 minutes. Cells were washed twice with PBS-BSA, and sorted on a BD FACS Aria II at the Roy J. Carver Biotechnology Center. The main cell population was gated by forward/side scattering to remove debris and doublets, and propidium iodide (1 μg/ml final) was added to the sample to exclude dead cells. Of the myc-positive (Alexa 647) population, the 15% of cells with the highest and lowest GFP fluorescence were collected (Fig 1F) in tubes coated overnight with fetal bovine serum and containing Expi293 Expression Medium. Total RNA was extracted from the collected cells using a GeneJET RNA purification kit (Thermo Scientific), and cDNA was reverse transcribed with high fidelity Accuscript primed with a gene-specific oligonucleotide (RevTrans_PDGFRA_992R, TCATGCAGGTTGACAGCTTC). The diversified region of PDGFRα was PCR amplified as 3 fragments to provide full coverage of the D2-D3 domains during Illumina sequencing. During PCR, flanking sequences on the primers added adapters to the ends of the products for annealing to Illumina sequencing primers, unique barcoding, and for binding the flow cell. Amplicons were sequenced on an Illumina NovaSeq 6000 using a 2×250 nt paired end protocol. Data were analyzed using Enrich [85], and commands are provided in the GEO deposit. Briefly, the frequencies of PDGFRα variants in the transcripts of the sorted populations were compared to their frequencies in the naive plasmid library to calculate an enrichment ratio.

### Production of soluble PDGFRα-Fc

Per ml of 2 × 10^6^ Expi293F cells, 500 ng of pcDNA3-sPDGFRα-Fc (IgG1) and 3 μg of polyethylenimine (MW 25,000; Polysciences) were mixed in 100 μl of OptiMEM (Gibco) and incubated for 20 minutes at room temperature prior to adding to cells. Transfection Enhancers were added 18 h post-transfection, and cells were cultured for six to seven days. Cells were removed by centrifugation at 600 × g for 20 minutes at 4°C. Cell debris and precipitates were removed by centrifugation at 18,000 × g for 25 minutes at 4°C. Supernatant was loaded on to KANEKA KanCapA 3G Affinity sorbent (Pall), and the resin was washed with PBS. Soluble PDGFRα-Fc was eluted with 60 mM sodium acetate pH 3.7, and 1 M Tris pH 8.0 was added to the eluate to neutralize the pH. Eluted sPDGFRα-Fc was concentrated with a centrifugal device (MWCO 100 kDa; Sartorius) and NaCl was added to 150 mM final. The protein was separated on a Superdex 200 Increase 10/300 GL (GE Healthcare Life Sciences) size exclusion chromatography column equilibrated with PBS. Peak fractions were pooled, concentrated, and stored at -80°C after snap freezing in liquid nitrogen. The proteins were quantified by absorbance at 280 nm using calculated molar extinction coefficients for the monomeric mature polypeptides.

Fusions of sPDGFRα to the Fc region of IgG3 were expressed as described above, and the expression medium was dialyzed against water. Cell debris was removed by centrifugation at 18,000 × g for 25 minutes at 4°C. Supernatant was loaded on to protein G HTC agarose beads (GoldBio) equilibrated with PBS. Protein was eluted with 100 mM glycine pH 2.5 and the eluate was neutralized by addition of 1 M Tris pH 9.0. Protein was further purified as described for the IgG1 fusions.

### Flow cytometry analysis of PDGFRα-Fc binding to HCMV trimer on the cell surface

400 ng of each of pcDNA3-gH (full-length), pcDNA3-gL-FLAG and pcDNA3-gO-8his were transfected in to Expi293F cells at 2 × 10^6^ / ml using Expifectamine. Transfection Enhancers were added 22 h later, and cells were harvested 46 h post-transfection. Cells were washed with PBS-BSA and incubated with PDGFRα-Fc (purified as described) or PDGFRβ-Fc (R&D Systems) for 40 minutes on ice. Cells were then washed 3 times with PBS-BSA and stained with anti-FLAG M2-Cy3 (Sigma), chicken anti-HIS-FITC (polyclonal, Immunology Consultants Laboratory), and anti-human IgG-APC (clone HP6017, BioLegend) for 30 minutes on ice. Cells were washed three times with PBS-BSA and analyzed by flow cytometry. To compare data across different experiments, the change in ΔMFU for each condition was normalized to the ΔMFU at the maximum concentration of WT sPDGFRα-Fc: Relative binding = (MFU_sample_−MFU_background_) / (MFU_max WT_ − MFU_background_).

### PDGFRα signaling assay

500 ng of PDGFRα reporter plasmid was transfected into 1 ml Expi293F cells at 2 × 10^6^ / ml using Expifectamine. 7.5 μl of 6 μM sPDGFRα-Fc (concentration based on monomer) and 7.5 μl of 2 μM PDGF were mixed, incubated at room temperature for 40 minutes, and then added to 1 ml cells at transfection. Human gamma globulin was from Jackson Immuno Research Labs, and PDGFs were from R&D Systems. Cells were collected 24 h post-transfection and stained with anti-myc-Alexa 647 to detect PDGFRα expression. GFP-PEST reporter expression was measured by flow-cytometry.

### Virus neutralization assays

Neutralization assays were performed similarly to Stegmann et al. [38] by serially diluting Fc-fused PDGFRα proteins or anti-HCMV immunoglobulin (Cytogam, CSL Behring) in PBS (vehicle) and incubating diluted proteins with HCMV virions for 1 h at 37°C. Virion-protein mixtures were adsorbed onto target cells for 2 h at 37°C / 5% CO_2_, washed once with PBS, and infected cells were allowed to recover for 18 h. The ability of each treatment to neutralize HCMV infection was measured using indirect-immunofluorescence by determining the number of cells expressing viral IE1, using anti-IE1 clone 1B12 [83] (generously provided by Thomas Shenk, Princeton University) and counterstaining nuclei with DAPI. A multiplicity of infection of 1 was used for AD169 infections, and a multiplicity of infection of 0.5 was used for TB40/E infections (based on stock titers acquired on MRC-5 fibroblasts).

### Structural modeling

The sequence of human PDGFRα was threaded onto the structure of PDGF-BB-bound PDGFRβ (PDB ID 3MJG [44]), with one of the two receptor chains and the D1 domain removed. The model was minimized with ROSETTA using FastRelax [86]. Connectivity was determined using the AverageDegree filter in ROSETTA [87]. Images were rendered with PyMOL (Schrödinger, LLC).

### Reagent and data availability

Plasmids are deposited with Addgene. Raw and processed deep sequencing data are deposited in NCBI’s Gene Expression Omnibus (GEO) under series accession number GSE138169.

## Supporting information

S1 Fig Binding of gO-sfGFP to PDGFRα-positive cells is dependent on co-expression of gH and gL. Expi293F cells were incubated with medium from cells expressing **(A)** gO-sfGFP, **(B)** gO(C343S)-sfGFP or **(C)** gH/gL/gO-sfGFP, washed and analyzed by flow cytometry. Cells were either untransfected (black), expressing PDGFRβ (blue) or expressing PDGFRα (red). High binding signal that was specific for PDGFRα-positive cells was only observed for medium containing all three components of the HCMV trimer.
(TIF)

S2 Fig A deep mutational scan of PDGFRα D2-D3 domains for HCMV trimer interactions in the presence of PDGFs. **(A)** Log_2_ enrichment ratios for individual mutations in the UL74-High sort are plotted from -3 (orange, depleted) to +3 (dark blue, enriched). Amino acid position is on the horizontal axis, and substitutions are on the vertical axis. *, stop codon. **(B)** Agreement between log_2_ enrichment ratios from independent replicates of the UL74-High sort (positive selection). R^2^ values are calculated for nonsynonymous mutations in black. Nonsense mutations are red. **(C)** Agreement between log_2_ enrichment ratios from replicates of the UL74-Low sort (negative selection). **(D)** Log_2_ enrichment ratios for nonsynonymous mutations (black) are anticorrelated between the negative and positive selections. Nonsense mutations (red) are depleted from both sorts due to lost surface expression. **(E-G)** High correlation between conservation scores (calculated by averaging the log_2_ enrichment ratios for all nonsynonymous mutations at a given amino acid position) from independent replicates of the UL74-High (E) and UL74-Low (F) sorts. Conservation scores are anticorrelated between the two sorted populations (G).
(TIF)

S3 Fig There are no hot spots for enriched mutations in the negative selection for loss of HCMV trimer binding. Log_2_ enrichment ratios for single amino acid substitutions of PDGFRα are plotted based on their enrichment in the UL74-Low sort, from -3 (orange, depleted) to +3 (dark blue, enriched). Amino acid position is on the horizontal axis, and substitutions are on the vertical axis. *, stop codon. Mutations to critical residues for HCMV trimer binding are anticipated to be enriched (dark blue) in this negative selection. However, there are no unambiguous hot spot regions for enriched mutations. Compare to the positive selection shown in S2A Fig, which uses the same color scale.
(TIF)

S4 Fig PDGFRα mutations that increase HCMV trimer binding in the presence of competing PDGFs are biased to structurally connected residues. Residue conservation scores in the UL74-High deep mutational scan were calculated by averaging the log_2_ enrichment ratios for all 20 possible amino acids at each diversified position. PDGFRα residues where mutations tend to increase HCMV trimer binding in the presence of competing PDGFs have higher positive scores. A residue’s conservation score is correlated with its connectivity in the modeled PDGF-bound PDGFRα structure, where connectivity is quantified by the number of neighboring residues within a 12 Å radius. Highly connected residues are either buried in the hydrophobic cores of the D2-D3 domains, or are buried at the PDGF binding interface.
(TIF)

S5 Fig Purification of soluble IgG1 Fc-fused PDGFRα. **(A)** The extracellular domain of PDGFRα (Gln24-Glu524; grey) was fused via a short linker (purple) to the Fc region of IgG1 (green). The “Legacy” sequence corresponds to the commercially supplied protein (R&D Systems) used in prior publications. The sequence was redesigned for this study. **(B)** Coomassie-stained SDS gel (run under denaturing and reducing conditions) of wild-type sPDGFRα-Fc eluted from a protein A column. The monomeric protein MW is predicted to be 82 kD. Additional weight may come from glycosylation and/or anomalous electrophoretic mobility. **(C)** SEC elution of wild-type (solid black line), Y206S (grey line) and V242K (dashed black line) sPDGFRα-Fc. UV absorbance (y-axis) is scaled.
(TIF)

S6 Fig Chemical stress tests of sPDGFRα-Fc. **(A)** The most promising engineered orthogonal receptor, sPDGFRα-Fc V242K, was incubated at 40°C for 7 days in 20 mM Tris pH 8.5 with 10 mM EDTA to promote Asn deamidation, or at 40°C for 14 days in 50 mM sodium acetate pH 5.5 to promote Asn isomerization. The control sample in PBS (pH 7.4) was flash frozen and stored at -80°C until analysis. SDS-polyacrylamide gel electrophoresis with Coomassie blue staining shows chemical instability of sPDGFRα-Fc V242K in the harsher pH 5.5 stress test. **(B)** Stressed proteins were analyzed by SEC on a Superdex 200 Increase 10/300 GL column with PBS pH 7.4 as the running buffer.
(TIF)

S7 Fig Soluble PDGFRα-Fc V242K binds HCMV trimer with comparable affinity to wild-type sPDGFRα-Fc. **(A)** Data presented in Fig 3D was replicated using independent preparations of sPDGFRα WT (solid black line) and V242K (broken black line) fused to the Fc region of IgG1. Binding to Expi293F cells expressing full-length gH, gL and gO from the HCMV TB40/E strain was assessed by flow cytometry. **(B and C)** Soluble PDGFRα WT (solid black line) and V242K (broken black line) were purified as fusions to the Fc region of IgG3, matching the redesigned linker described in S5A Fig. Binding to trimer from (B) TB40/E and (C) Merlin strains expressed on Expi293F cells was measured by flow cytometry. Data are mean ± SD, n = 3 (sPDGFRα-Fc WT and V242K) or 2 (sPDGFRβ-Fc).
(TIF)

S8 Fig Gamma globulin has no effect on PDGF signaling in culture. PDGF signaling was assessed in Expi293F cells transiently transfected with a PDGFRα reporter plasmid as outlined in Fig 4A. A 3-fold molar excess of wild-type sPDGFRα-Fc (black) blocks 5 nM PDGF-AB signaling. Orthogonal engineered sPDGFRα-Fc V242K (white) does not block signaling and the mutation shows no interactions with PDGFs in competitive binding experiments (Fig 3). Furthermore, wild-type or mutant sPDGFRα-Fc up to 100 nM shows no binding to PDGFRα-positive cells, and we exclude unanticipated interactions between receptor chains. However, sPDGFRα-Fc V242K does cause an increase in signaling of PDGF-A ligands (AA and AB) for unknown reasons. A non-specific carrier effect in which the orthogonal receptor stabilizes the hydrophobic ligand in solution is suspected, especially because the more hydrophobic variant sPDGFRα-Fc Y206S promotes an even bigger increase in PDGF-A signaling (Fig 4B). Human gamma globulin (violet) at the same concentration has no effect in this assay. Data are mean ± SD, n = 2 independent replicates.
(TIF)
