# Supplementary figures and images for: Engineered receptors for human cytomegalovirus that are orthogonal to normal human biology
Source: PLoS Pathog. 2020 Jun 19;16(6):e1008647. doi: 10.1371/journal.ppat.1008647 (PMC7329128; doi:10.1371/journal.ppat.1008647)

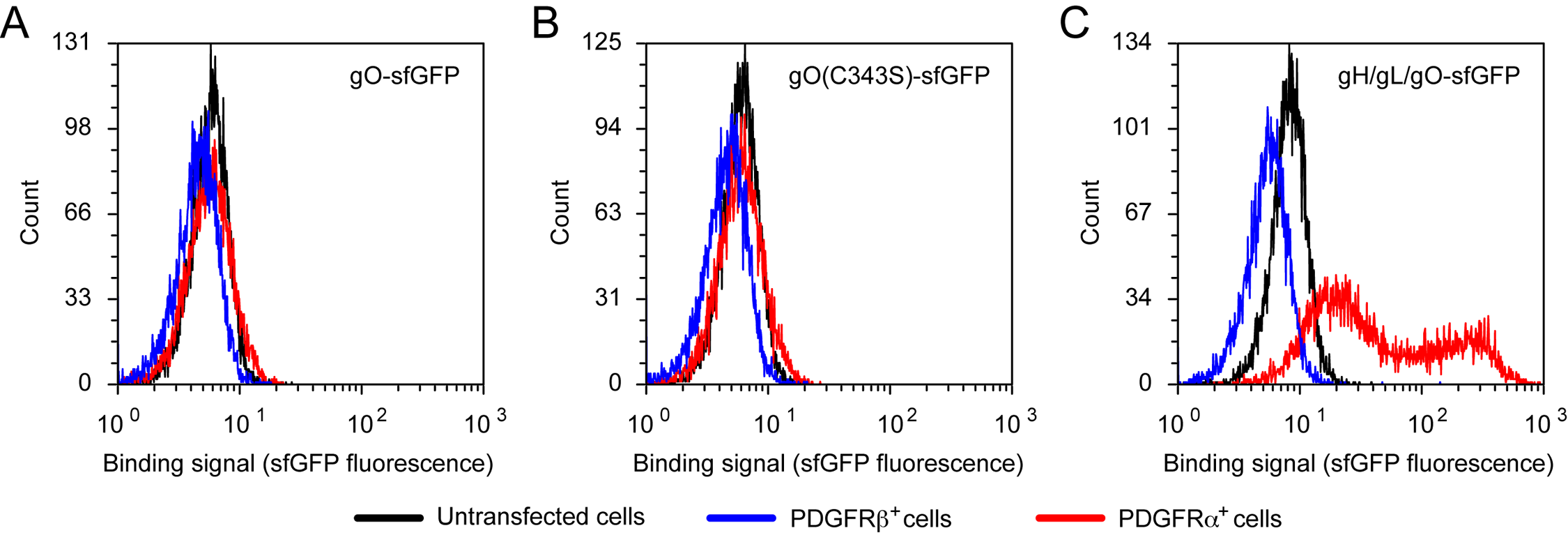

Supplement: S1 Fig — Expi293F cells were incubated with medium from cells expressing (A) gO-sfGFP, (B) gO(C343S)-sfGFP or (C) gH/gL/gO-sfGFP, washed and analyzed by flow cytometry. Cells were either untransfected (black), expressing PDGFRβ (blue) or expressing PDGFRα (red). High binding signal that was specific for PDGFRα-positive cells was only observed for medium containing all three components of the HCMV trimer. (TIF) [file ppat.1008647.s001.tif]

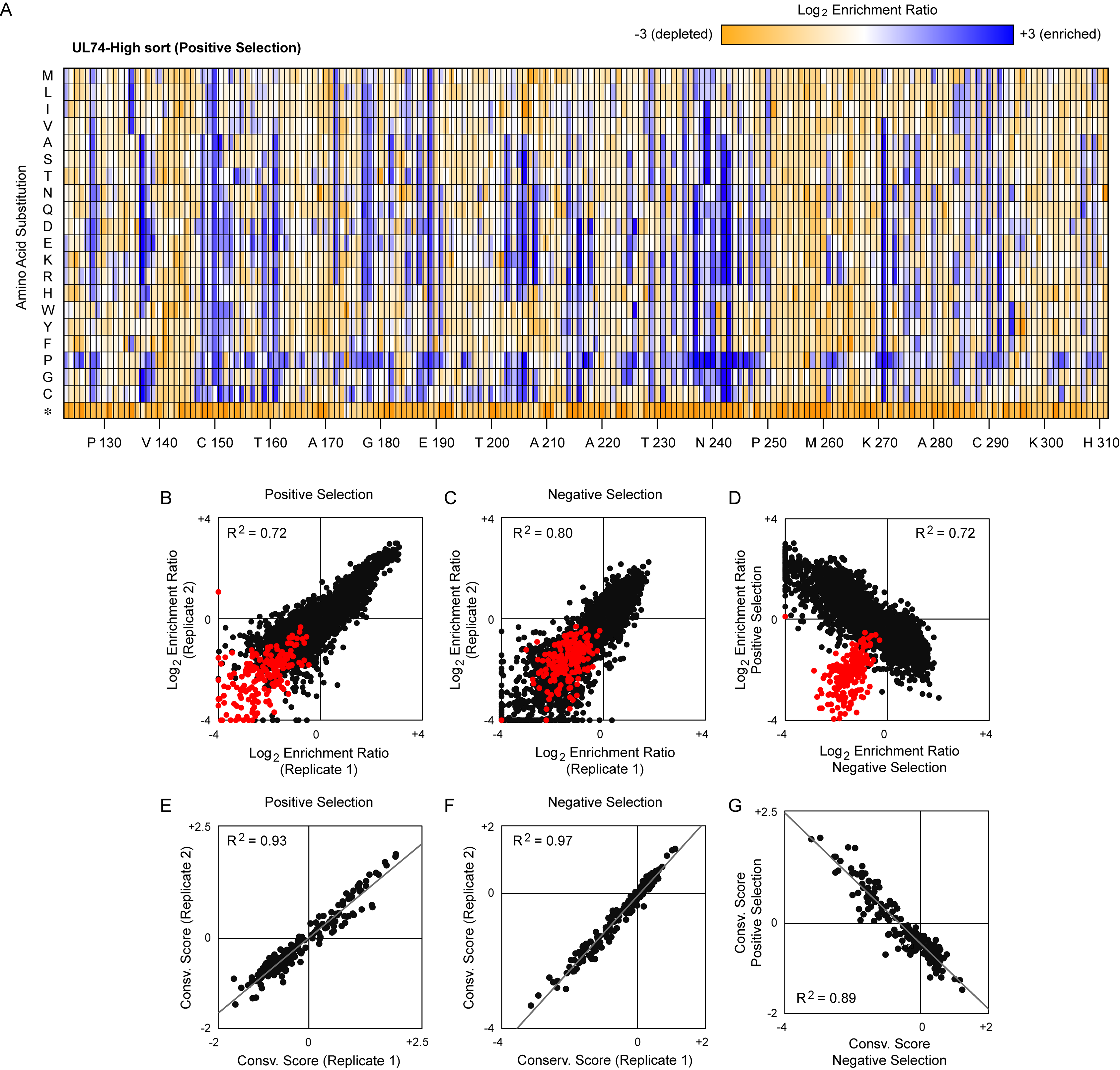

Supplement: S2 Fig — (A) Log2 enrichment ratios for individual mutations in the UL74-High sort are plotted from -3 (orange, depleted) to +3 (dark blue, enriched). Amino acid position is on the horizontal axis, and substitutions are on the vertical axis. *, stop codon. (B) Agreement between log2 enrichment ratios from independent replicates of the UL74-High sort (positive selection). R2 values are calculated for nonsynonymous mutations in black. Nonsense mutations are red. (C) Agreement between log2 enrichment ratios from replicates of the UL74-Low sort (negative selection). (D) Log2 enrichment ratios for nonsynonymous mutations (black) are anticorrelated between the negative and positive selections. Nonsense mutations (red) are depleted from both sorts due to lost surface expression. (E-G) High correlation between conservation scores (calculated by averaging the log2 enrichment ratios for all nonsynonymous mutations at a given amino acid position) from independent replicates of the UL74-High (E) and UL74-Low (F) sorts. Conservation scores are anticorrelated between the two sorted populations (G). (TIF) [file ppat.1008647.s002.tif]

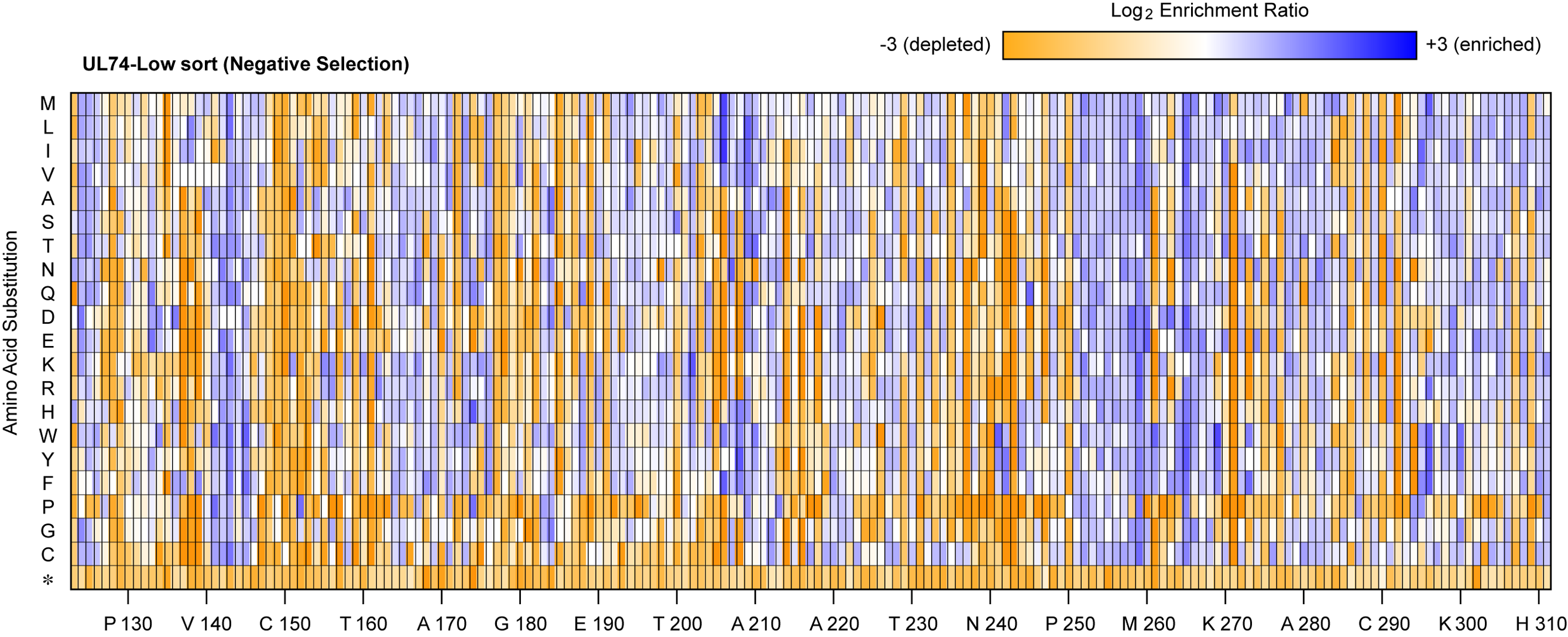

Supplement: S3 Fig — Log2 enrichment ratios for single amino acid substitutions of PDGFRα are plotted based on their enrichment in the UL74-Low sort, from -3 (orange, depleted) to +3 (dark blue, enriched). Amino acid position is on the horizontal axis, and substitutions are on the vertical axis. *, stop codon. Mutations to critical residues for HCMV trimer binding are anticipated to be enriched (dark blue) in this negative selection. However, there are no unambiguous hot spot regions for enriched mutations. Compare to the positive selection shown in S2A Fig, which uses the same color scale. (TIF) [file ppat.1008647.s003.tif]

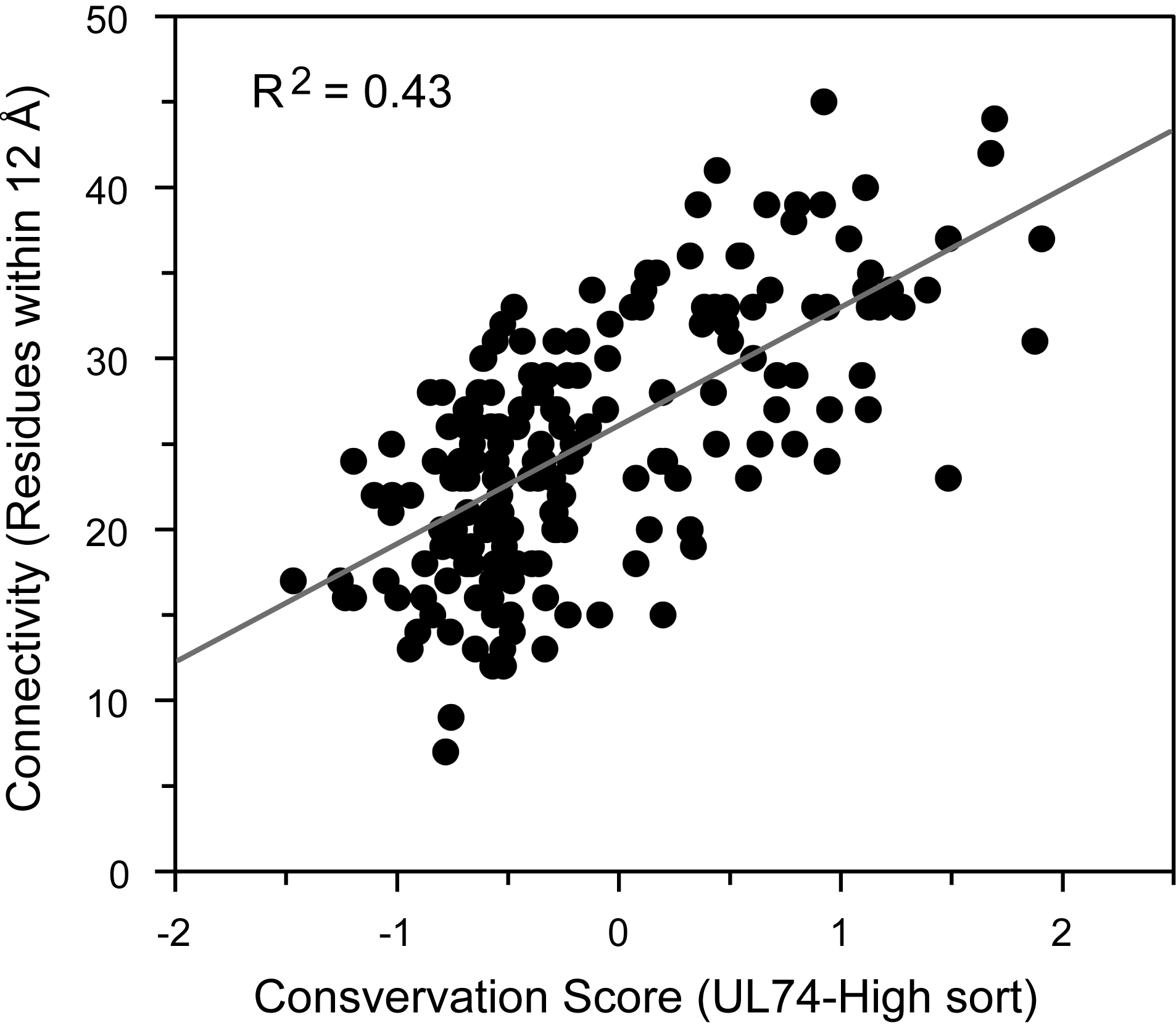

Supplement: S4 Fig — Residue conservation scores in the UL74-High deep mutational scan were calculated by averaging the log2 enrichment ratios for all 20 possible amino acids at each diversified position. PDGFRα residues where mutations tend to increase HCMV trimer binding in the presence of competing PDGFs have higher positive scores. A residue’s conservation score is correlated with its connectivity in the modeled PDGF-bound PDGFRα structure, where connectivity is quantified by the number of neighboring residues within a 12 Å radius. Highly connected residues are either buried in the hydrophobic cores of the D2-D3 domains, or are buried at the PDGF binding interface. (TIF) [file ppat.1008647.s004.tif]

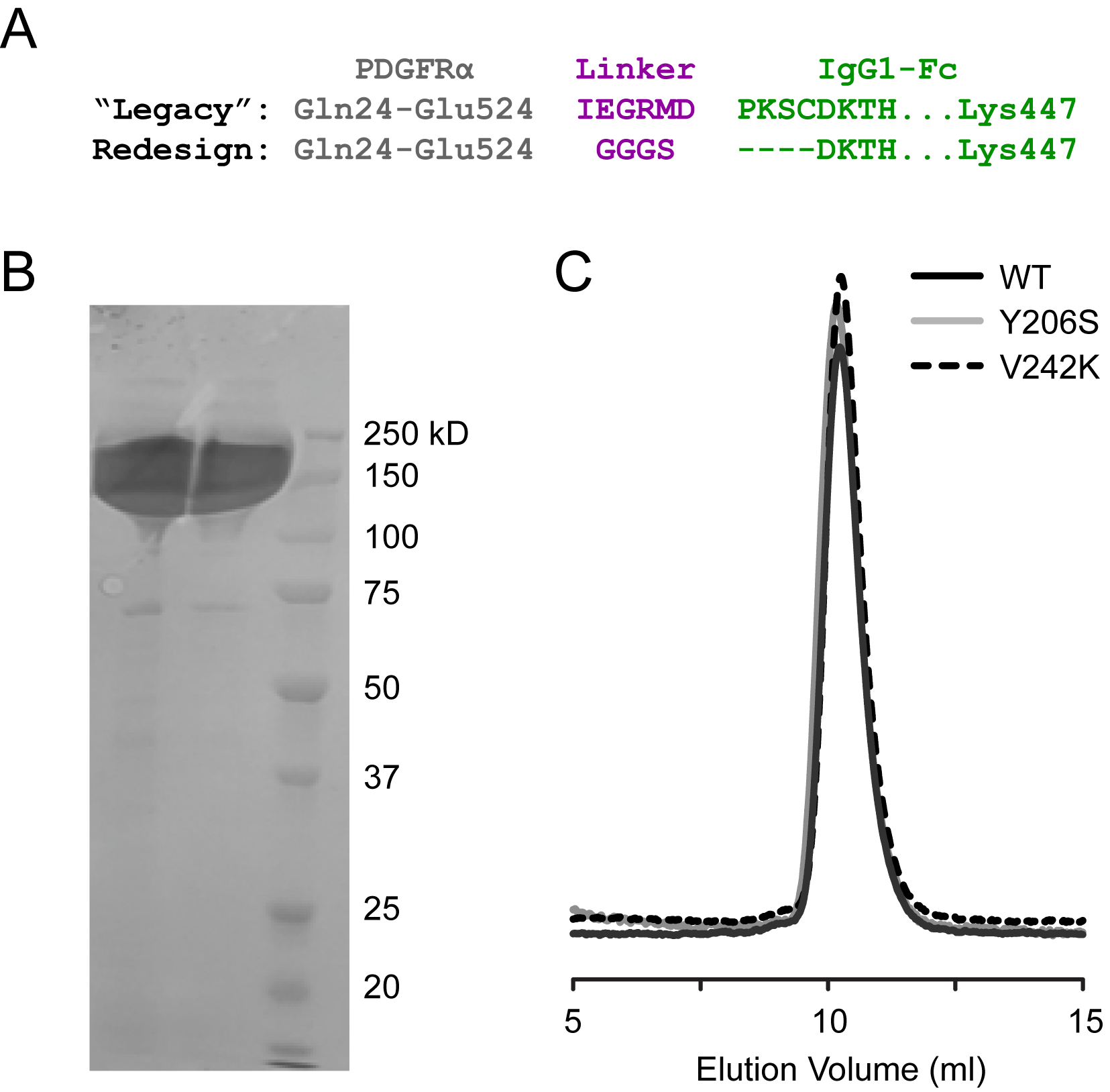

Supplement: S5 Fig — (A) The extracellular domain of PDGFRα (Gln24-Glu524; grey) was fused via a short linker (purple) to the Fc region of IgG1 (green). The “Legacy” sequence corresponds to the commercially supplied protein (R&D Systems) used in prior publications. The sequence was redesigned for this study. (B) Coomassie-stained SDS gel (run under denaturing and reducing conditions) of wild-type sPDGFRα-Fc eluted from a protein A column. The monomeric protein MW is predicted to be 82 kD. Additional weight may come from glycosylation and/or anomalous electrophoretic mobility. (C) SEC elution of wild-type (solid black line), Y206S (grey line) and V242K (dashed black line) sPDGFRα-Fc. UV absorbance (y-axis) is scaled. (TIF) [file ppat.1008647.s005.tif]

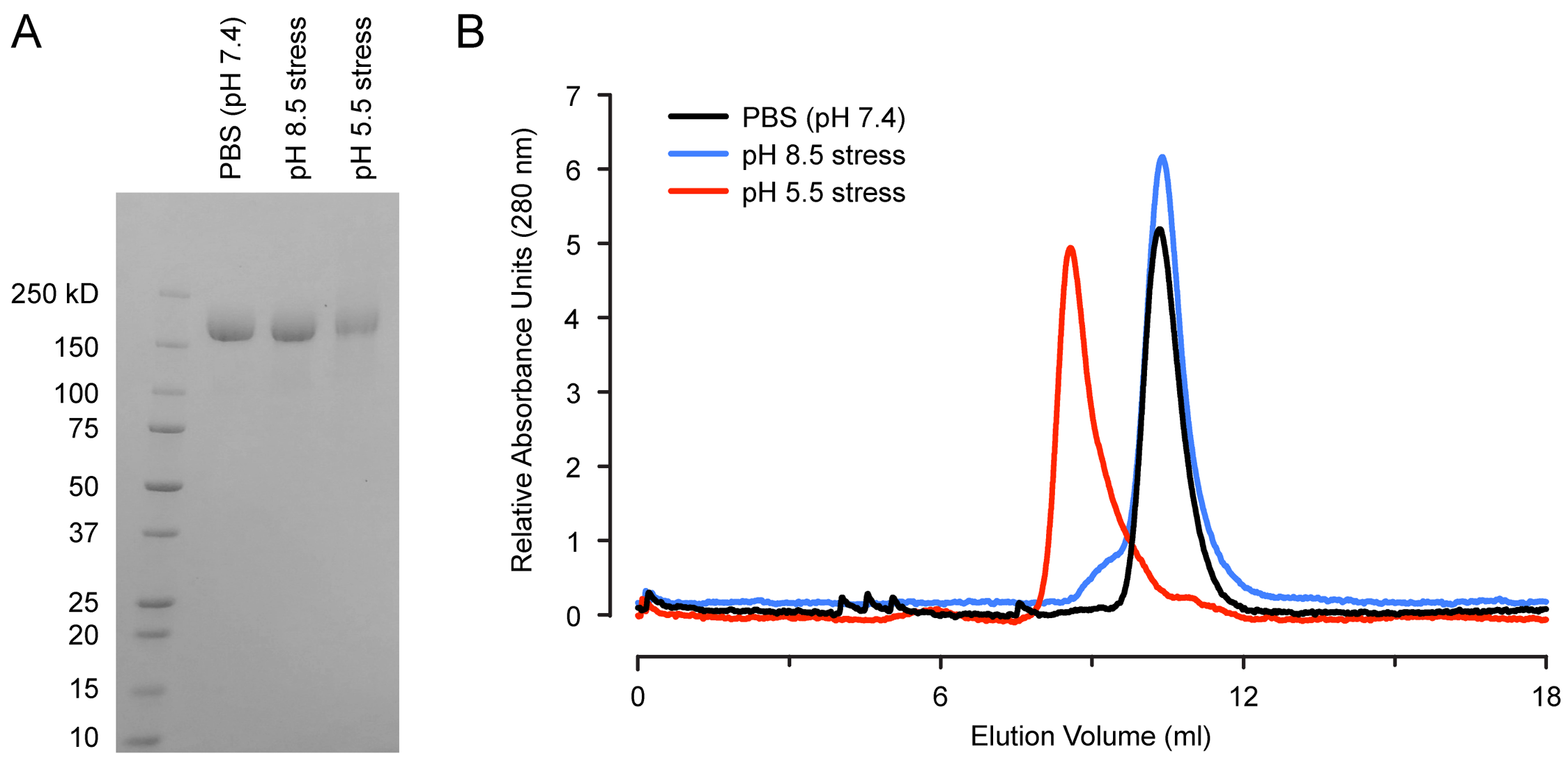

Supplement: S6 Fig — (A) The most promising engineered orthogonal receptor, sPDGFRα-Fc V242K, was incubated at 40°C for 7 days in 20 mM Tris pH 8.5 with 10 mM EDTA to promote Asn deamidation, or at 40°C for 14 days in 50 mM sodium acetate pH 5.5 to promote Asn isomerization. The control sample in PBS (pH 7.4) was flash frozen and stored at -80°C until analysis. SDS-polyacrylamide gel electrophoresis with Coomassie blue staining shows chemical instability of sPDGFRα-Fc V242K in the harsher pH 5.5 stress test. (B) Stressed proteins were analyzed by SEC on a Superdex 200 Increase 10/300 GL column with PBS pH 7.4 as the running buffer. (TIF) [file ppat.1008647.s006.tif]

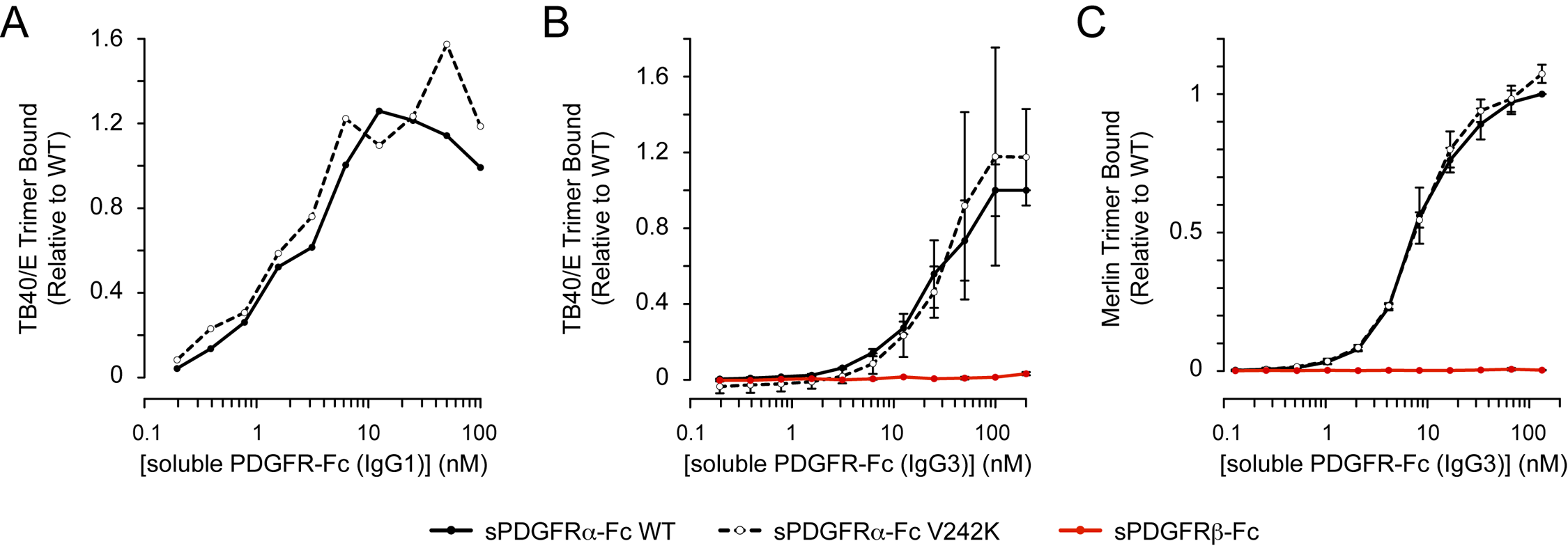

Supplement: S7 Fig — (A) Data presented in Fig 3D was replicated using independent preparations of sPDGFRα WT (solid black line) and V242K (broken black line) fused to the Fc region of IgG1. Binding to Expi293F cells expressing full-length gH, gL and gO from the HCMV TB40/E strain was assessed by flow cytometry. (B and C) Soluble PDGFRα WT (solid black line) and V242K (broken black line) were purified as fusions to the Fc region of IgG3, matching the redesigned linker described in S5A Fig. Binding to trimer from (B) TB40/E and (C) Merlin strains expressed on Expi293F cells was measured by flow cytometry. Data are mean ± SD, n = 3 (sPDGFRα-Fc WT and V242K) or 2 (sPDGFRβ-Fc). (TIF) [file ppat.1008647.s007.tif]

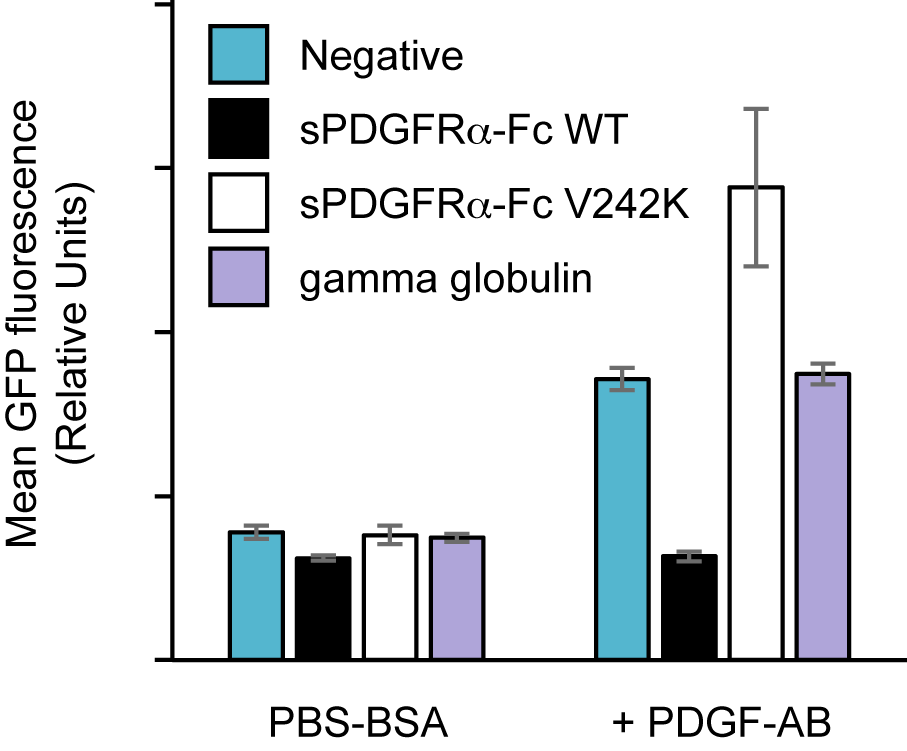

Supplement: S8 Fig — PDGF signaling was assessed in Expi293F cells transiently transfected with a PDGFRα reporter plasmid as outlined in Fig 4A. A 3-fold molar excess of wild-type sPDGFRα-Fc (black) blocks 5 nM PDGF-AB signaling. Orthogonal engineered sPDGFRα-Fc V242K (white) does not block signaling and the mutation shows no interactions with PDGFs in competitive binding experiments (Fig 3). Furthermore, wild-type or mutant sPDGFRα-Fc up to 100 nM shows no binding to PDGFRα-positive cells, and we exclude unanticipated interactions between receptor chains. However, sPDGFRα-Fc V242K does cause an increase in signaling of PDGF-A ligands (AA and AB) for unknown reasons. A non-specific carrier effect in which the orthogonal receptor stabilizes the hydrophobic ligand in solution is suspected, especially because the more hydrophobic variant sPDGFRα-Fc Y206S promotes an even bigger increase in PDGF-A signaling (Fig 4B). Human gamma globulin (violet) at the same concentration has no effect in this assay. Data are mean ± SD, n = 2 independent replicates. (TIF) [file ppat.1008647.s008.tif]
